# Primary care physicians’ attitude and reported prescribing behavior for chronic low back pain: An exploratory cross-sectional study

**DOI:** 10.1371/journal.pone.0204613

**Published:** 2018-09-27

**Authors:** Pierre-Yves Rodondi, Julie Dubois, Anne-Sylvie Bill, Daria Koutaïssoff, Jenny Ros, Eléonore Aveni, Jérôme Pasquier, Lilli Herzig, Isabelle Decosterd, Bernard Burnand

**Affiliations:** 1 Institute of Social and Preventive Medicine, Lausanne University Hospital and Faculty of Biology and Medicine, University of Lausanne, Lausanne, Switzerland; 2 Institute of Family Medicine, University of Fribourg, Fribourg, Switzerland; 3 Institute of Family Medicine, University of Lausanne, Lausanne, Switzerland; 4 Pain Center and Center for integrative and complementary medicine, Department of anesthesiology, Lausanne University Hospital and Faculty of Biology and Medicine, University of Lausanne, Lausanne, Switzerland; The University of Sydney, AUSTRALIA

## Abstract

**Objective:**

Recent guidelines for chronic or recurrent low back pain recommend non-pharmacologic treatments as first-line options. The objective of this study was thus to explore the perceived usefulness of several conventional and complementary medicine treatments for chronic or recurrent low back pain by primary care physicians and their reported prescribing behavior.

**Design:**

An exploratory cross-sectional study.

**Setting and participants:**

Primary care physicians of the French-speaking part of Switzerland.

**Main outcome measures:**

Primary care physicians’ perceived usefulness of each conventional and complementary medicine treatment and their reported recommendation behavior were considered dependent variables in multivariate logistic regression models. All correlations were computed between binary variables, and phi coefficients were calculated to estimate correlation strengths.

**Results:**

533 primary care physicians answered the questionnaire (response rate: 25.6%). The top 3 conventional treatments most often considered useful by primary care physicians for chronic or recurrent low back pain were physiotherapy (94.8%), nonsteroidal anti-inflammatory drugs (87.9%), and manual therapy (82.5%), whereas the most prescribed conventional treatments were physiotherapy (99.2%), nonsteroidal anti-inflammatory drugs (97.4%), and acetaminophen (94.4%). Osteopathic treatment (78.4%), yoga (69.3%), and therapeutic massage (63.9%) were the complementary medicine treatments most often considered useful by primary care physicians in managing chronic or recurrent low back pain. Being a female physician, younger than 56 years, trained in complementary medicine, or using complementary medicine were all associated with higher perceived usefulness of complementary medicine treatments in general. The most recommended complementary medicine treatments by primary care physicians were osteopathic treatment (87.3%), acupuncture (69.3%), and therapeutic massage (58.7%). Being a female physician, younger than 56, and using complementary medicine were all associated with more complementary medicine recommendation in general.

**Conclusion:**

Our results highlight the importance of better understanding the prescribing patterns of primary care physicians for chronic or recurrent low back pain. Considering the frequency and burden of chronic or recurrent low back pain, programs focusing on the most (cost-) effective treatments should be implemented.

## Introduction

Chronic low back pain is a common condition, with a global prevalence situated around 20% [1]. Furthermore, recurrent low back pain is also common, with 33% recurrence within the first year following an acute episode of low back pain (LBP) [2]. Conventional treatment options for chronic or recurrent LBP include drug therapies, nonmedical interventions, and surgery [3]. According to a recent systematic review and meta-analysis including 35 randomized placebo-controlled trials, non-steroidal anti-inflammatory drugs (NSAIDs) reduced pain and disability in the immediate and short-term, but did not have clinically important effects on pain intensity [4]. Regarding opioids, another systematic review and meta-analysis showed a short-term effect on pain, but the effect was small and probably not clinically relevant [5]. When it comes to non-pharmacologic interventions, exercise therapy reduced pain intensity and disability better than usual care and behavioral therapies had an effect on pain intensity [6]. Additionally, current scientific data available about complementary medicine (CM) is increasing. The World Health Organization (WHO) defines CM as “a broad set of health care practices that are not part of that country’s own tradition or conventional medicine and are not fully integrated into the dominant health-care system”. Some studies suggest that CM non-pharmacologic therapies such as acupuncture [7–9], yoga [10, 11], Tai-Chi [12], Mindfulness-based stress reduction [13], osteopathy [14] and hypnosis [15] might be useful treatment options for patients suffering from chronic LBP [16], although more studies are needed.

Available guidelines regarding the treatment of chronic or recurrent LBP tend to be similar from one country to another [17], and include the initial first-line short-term prescription of medication (acetaminophen and NSAIDs), followed by non-pharmacologic therapies such as cognitive behavioral therapy or some CM, including therapeutic massage, relaxation, yoga, spinal manipulation, and acupuncture[18]. However, the 2017 American College of Physicians guidelines changed the overall paradigm by recommending non-pharmacologic treatments as first-line options [19]. Conventional drug treatments (NSAIDs, opioids, and duloxetine) were to be prescribed only if the first-line treatment failed, with acetaminophen being no longer included in the guidelines, based on a a recent meta-analysis [20]. It is worthy to note that the recent UK guidelines also emphasized on non-pharmacologic treatments for chronic LBP, including some CM if they were part of an exercise program [21]. However, contrary to the American College of Physicians guidelines, the UK guidelines did not recommend acupuncture [22]. They also did not discriminate between first- and second-line treatments.

Practical implementation of guidelines remains a challenge, despite a tremendous amount of scientific literature available to physicians [17]. Although widely disseminated, clinical practice guidelines have limited effect in changing physicians’ behavior [23–26]. Lack of agreement with recommendations among primary care physicians (PCPs), lack of applicability due to the heterogeneity of the patient population, various perceptions of treatment effectiveness, PCPs’ own professional and personal experience, patients’ pressure or perceived pressure, patients’ preferences, and environmental factors appeared to be some of the main barriers to the implementation of guidelines in practice [27–36]. Furthermore, physicians’ beliefs about low back pain management may influence treatment choices [25, 37]. Given the multiple factors influencing the management of chronic or recurrent LBP, it is important to evaluate treatment preferences and attitudes of PCPs, all the more so as to our knowledge there have been no studies associating treatment preferences of PCPs with their perceived usefulness of these treatments since 1995 [38].

The objective of this study was thus to explore the perceived usefulness of several treatments for chronic or recurrent LBP by PCPs and their reported prescribing behavior, including both conventional and CM options. The factors associated with perceived usefulness and reported prescribing behavior were also explored.

## Methods

### Design, setting, and participants

This exploratory cross-sectional study was conducted by means of a postal questionnaire sent to all PCPs practicing in the French-speaking part of Switzerland (n = 2443). Postal addresses of PCPs were obtained from the local medical societies, and their socio-demographic data from the Swiss Medical Association (FMH). The number of PCPs registered in the local medical societies (n = 2443) differed from those registered in FMH (n = 1757) for 2 possible reasons: there were a high number of false addresses among the local medical societies; and the FMH data included PCPs working outside hospitals only, whereas the data from the local medical societies also included PCPs working inside hospitals. Finally, 2085 questionnaires were sent by post in April 2016, and a reminder was sent 3 weeks later.

### Variables

The questionnaire included 79 items and was divided into 4 parts: (1) socio-demographic data, (2) PCPs’ perceived usefulness of various conventional treatments for chronic or recurrent LBP and their reported prescribing behavior, (3) PCPs’ perceived usefulness of CM in general and of specific CM treatments and their recommendations, and (4) PCP’s attitude towards and personal use of CM. Perceived usefulness of conventional and CM treatment for chronic or recurrent LBP was compared with reported prescribing or recommendation behavior of PCPs. Perceived usefulness was defined by means of a categorical question asking whether or not physicians agreed that one therapy could be useful in the treatment of chronic or recurrent LBP (see statistical analysis). Reported prescription rates of conventional treatments and recommendations for CM corresponded to the percentage of PCPs who reported having prescribed or recommended any given treatment option at least once to a patient with chronic or recurrent LBP.

### Data Sources

In the absence of a validated questionnaire, we built one based on a literature review. Some questions were also adapted from two existing questionnaires [39, 40], one on patients’ perceived usefulness of treatments for LBP and the other on physicians’ attitude toward complementary medicine. The study questionnaire was piloted and tested in 10 PCPs.

According to the International Association for the Study of Pain, chronic low back pain is characterized as pain lasting or recurring for three months or more [41]. For this study, recurrent low back pain was defined as two episodes or more of low back pain during the past year, with a significant impact on the patient’s daily life. This definition was built on a consensus of 15 PCPs, researchers and pain specialists. The Cantonal Commission for the Ethics of Human Research (CER-VD) approved the study (Ref: 303/15). The ethics committee waived the need for consent, as data were collected and analyzed anonymously.

### Statistical analysis

Wilson confidence intervals were computed for all proportions [42]. For each conventional and CM treatment, we fitted a multivariate logistic regression model to evaluate the associations between perceived usefulness and the following variables: age (<56 years old, ≥56 years old), gender (male, female), CM training (no, yes), and CM use (no, yes). We did the same for recommendation behavior instead of perceived usefulness. As we considered 29 different treatments and CM treatments in general, we fitted a total of 60 logistic regression models. The models were constructed as follows: the perceived usefulness and reported recommendation behavior were considered as the dependent variable, and age, gender, CM training, and CM use as the independent variables. For the regression models examining variables associated to perceived usefulness we dichotomized usefulness scale (5 levels) as follows: "strongly disagree", "disagree" and "neither agree nor disagree" were recoded as "not useful", while "agree" and "strongly agree" were recoded as "useful". For the regression models examining variables associated to reported recommendation we dichotomized the reported percentage of patients (6 possibilities) to whom a recommendation was done as follow: "0%" were recoded as "do not recommend" while "1–100%" were recoded as "recommend". In models in which the dependent variable (perceived usefulness or reported recommendation behavior) was related to a conventional treatment, we considered CM use in general as an independent variable. On the other hand, in models in which the dependent variable was related to a CM treatment, we considered the use of the corresponding CM as an independent variable. All correlations were computed between binary variables, and therefore phi coefficients were calculated to estimate correlation strengths. The missing values were imputed 20 times by the method of multiple imputation by chained equations. All variables which were used in the analyses were involved in the imputation models. All results are based on the multiple imputation. Data were entered into an Access database and analyzed with R statistical software (version 3.4.2). Missing values were handled with the “mice package” (version 2.30) [43].

## Results

### Socio-demographic data

**Fig 1:**
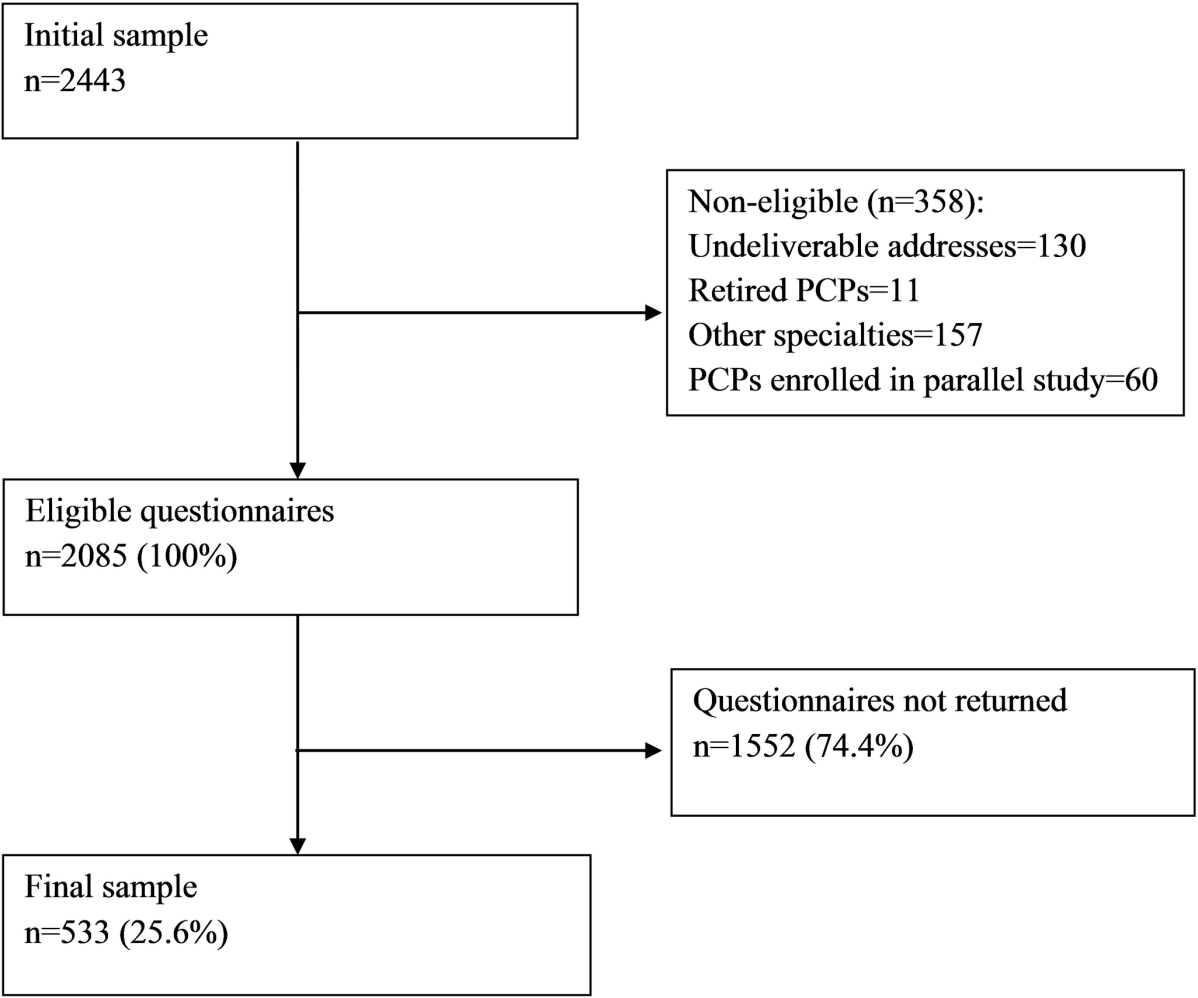
Diagram of study participation. PCPs = primary care physicians.

Of the 2443 questionnaires sent, 1552 were not returned and 358 were non-eligible. The final sample consisted of 533 questionnaires available for analysis (response rate: 25.6%). Participants did not always respond to all questions: 442 (82.9%) had no missing values among the variables used for the analyses. However, the missing rate per variable did not exceed 3.6%. Fig 1 shows the study participation.

Among respondents, 323 were men, 196 were women, and 14 did not disclose their gender. In comparison to the overall PCPs practicing in the French part of Switzerland [44], the representativeness of our sample was good in terms of gender, age, and training in CM. Table 1 shows the socio-demographic data of the respondents.

**Table 1:** Socio-demographic data of survey respondents compared with all primary care physicians (PCPs) in the French part of Switzerland.

	Survey respondents (n = 533)	All primary care physicians in the French part of Switzerland (n = 1757)^†^
**Gender (n = 519)**		
Men	323 (62.2%)	1143 (65.1%)
Women	196 (37.8%)	614 (34.9%)
**Age (n = 528)**		
≤55 years old	286 (54.2%)	843 (48.0%)
≥56 years old	242 (45.8%)	914 (52.0%)
**Training in complementary medicine (n = 533)**		
Yes No	127 (23.8%)^‡^ 406 (76.2%)	239 (13.6%)^§^
**Personal use of complementary medicine (n = 518)**		
Yes No	287 (55.4%) 231 (44.6%)	Not available

Results are expressed as number of PCPs (percentage).

^†^ Data of the Swiss Medical Association (FMH) for 2015.

^‡^ PCPs with or without a complementary medicine training certificate delivered by the FMH.

^§^ Only PCPs with a complementary medicine training certificate delivered by the FMH.

### Perceived usefulness of conventional treatments

**Fig 2:**
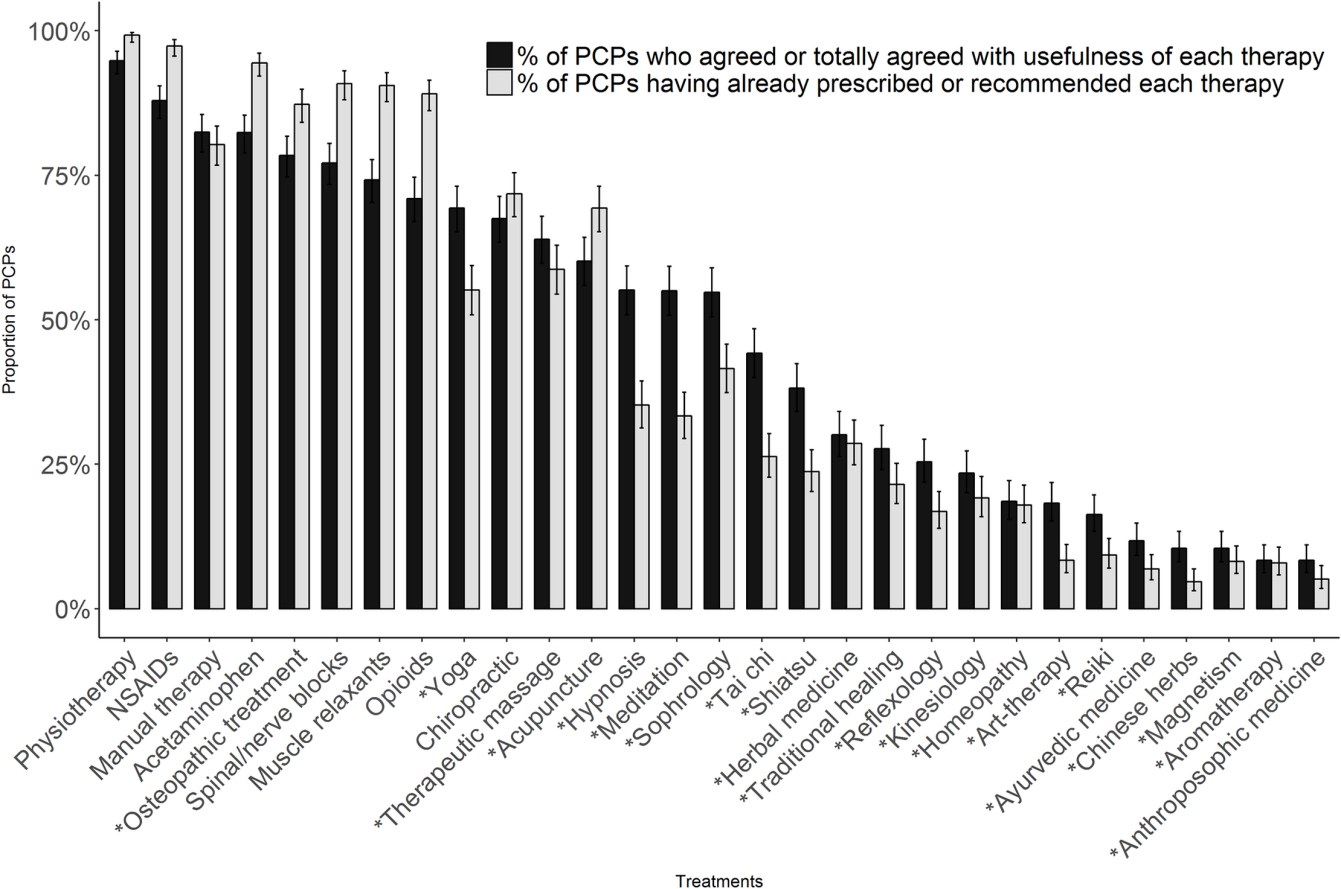
Perceived usefulness of chronic or recurrent LBP treatments among PCPs compared with reported prescribing/recommendation behavior. For each treatment, the proportion of PCPs who agree with its usefulness (dark gray) or have already prescribed or recommended it (gray) is shown. Error bars express 95% confidence interval. chronic or recurrent LBP = chronic or recurrent low back pain; PCPs = primary care physicians; NSAIDs = nonsteroidal anti-inflammatory drugs. *Complementary medicine.

Conventional therapies most often considered useful by PCPs for the treatment of chronic or recurrent LBP were: physiotherapy (94.8% [95% confidence interval (CI), 92.5–96.4%]), NSAIDs (87.9% [95% CI, 84.9–90.4%]), and manual therapy (82.5% [95% CI, 79.0–85.5%]). Conventional treatments perceived as being less useful were spinal/nerve blocks (77.1% [95% CI, 73.4–80.5%]), muscle relaxants (74.2% [95% CI, 70.3–77.7%]), and opioids (70.9% [95% CI, 67.0–74.6%]). Fig 2 shows a comparison between PCPs’ perceived usefulness of conventional and CM treatments and their reported prescribing behavior (in that figure, treatments considered as CM are indicated by an asterisk).

In a multivariate analysis, we did not observe a clear influence of gender, age or personal use of CM on perceived usefulness of conventional treatments. However, there was a tendency for PCPs with CM training to find conventional treatments less useful than PCPs without such training. Table 2 shows the perceived usefulness of both conventional and CM treatments by gender, age, training in CM, and personal use of CM.

**Table 2:** Multivariate regression models of perceived usefulness of conventional and complementary medicine treatments.

Treatments	Gender	CM training	Age	Personal use of CM ^¶^
	Female^†^	Yes^‡^	≥56 years^§^	Yes^I^
	OR (95% CI)	OR (95% CI)	OR (95% CI)	OR (95% CI)
**Conventional treatments**				
Physiotherapy	1.4 (0.6–3.5)	0.6 (0.2–1.5)	1.1 (0.5–2.6)	1.4 (0.6–3.3)
NSAIDs	1.4 (0.7–2.5)	0.7 (0.4–1.3)	0.6 (0.4–1.1)	0.6 (0.3–1.1)
Manual therapy	1.3 (0.8–2.3)	1.0 (0.6–1.8)	0.5 (0.3–0.8)**	1.9 (1.1–3.1)*
Acetaminophen	0.6 (0.4–1.0)	0.6 (0.4–1)	0.7 (0.4–1.2)	1.0 (0.6–1.6)
Spinal/nerve blocks	0.9 (0.6–1.5)	0.5 (0.3–0.8)**	0.8 (0.5–1.2)	0.6 (0.4–1.0)
Muscle relaxants	1.7 (1.1–2.6)*	1.0 (0.6–1.7)	1.0 (0.7–1.6)	1.0 (0.7–1.6)
Opioids	0.7 (0.4–1.0)*	0.5 (0.3–0.8)**	0.7 (0.5–1.0)	1.0 (0.6–1.5)
Chiropractic	0.7 (0.5–1.1)	0.8 (0.5–1.2)	1.1 (0.7–1.6)	1.2 (0.8–1.8)
**Complementary medicine**				
Osteopathic treatment	1.1 (0.7–1.8)	1.1 (0.6–1.9)	0.6 (0.4–1.0)*	7.0 (3.5–13.9)***
Yoga	2.3 (1.5–3.6)***	1.7 (1.0–2.7)*	0.7 (0.5–1.0)	17.3 (2.3–128.1)**
Therapeutic massage	1.2 (0.8–1.8)	1.8 (1.1–2.8)*	1.0 (0.7–1.5)	4.5 (2.2–9.4)***
Acupuncture	2.1 (1.4–3.2)***	1.7 (1.1–2.8)*	0.7 (0.5–1.1)	3.3 (1.9–5.8)***
Hypnosis	1.7 (1.2–2.6)**	1.6 (1.0–2.4)*	0.6 (0.4–0.9)*	3.9 (1.7–9.2)**
Meditation	2.1 (1.4–3.1)***	1.6 (1.0–2.5)*	0.5 (0.3–0.7)***	6.9 (2.8–16.9)***
Sophrology	1.7 (1.2–2.5)**	1.5 (1.0–2.3)	1.1 (0.7–1.6)	7.7 (2.7–22.3)***
Tai chi	2.3 (1.6–3.4)***	1.9 (1.2–2.9)**	0.7 (0.5–1.0)	10.3 (3.0–35.2)***
Shiatsu	1.5 (1.0–2.3)*	2.4 (1.5–3.7)***	1.0 (0.7–1.5)	12.6 (2.8–55.6)***
Herbal medicine	1.1 (0.7–1.7)	2.0 (1.3–3.1)**	0.8 (0.5–1.2)	3.1 (1.8–5.1)***
Traditional healing	1.0 (0.7–1.6)	1.8 (1.2–2.9)**	1.4 (0.9–2.1)	7.5 (2.8–19.9)***
Reflexology	1.5 (1.0–2.3)	2.5 (1.6–4.0)***	0.9 (0.6–1.4)	8.1 (3.5–18.9)***
Kinesiology	1.1 (0.7–1.8)	1.6 (1.0–2.5)	1.2 (0.8–1.9)	6.2 (2.3–16.3)***
Homeopathy	1.8 (1.1–3.0)*	2.4 (1.4–4.2)**	1.0 (0.6–1.7)	5.6 (3.2–9.8)***
Art-therapy	2.0 (1.2–3.2)**	1.2 (0.7–2.1)	0.6 (0.4–1.0)*	8.3 (1.4–50.7)*
Reiki	1.5 (0.9–2.5)	1.4 (0.8–2.4)	0.7 (0.4–1.2)	6.0 (2.5–14.7)***
Ayurvedic medicine	2.0 (1.1–3.7)*	3.5 (1.9–6.4)***	0.6 (0.3–1.1)	10.9 (3.8–31.3)***
Chinese herbs	1.2 (0.7–2.2)	4.2 (2.3–7.8)***	0.6 (0.3–1.2)	5.7 (1.9–17.3)**
Magnetism	1.5 (0.8–2.9)	3.5 (1.8–6.8)***	1.1 (0.6–2.2)	28.2 (10–79.3)***
Aromatherapy	1.1 (0.5–2.3)	3.1 (1.5–6.4)**	0.7 (0.3–1.5)	9.6 (4.2–21.8)***
Anthroposophic medicine	2.3 (1.2–4.4)*	4.2 (2.2–8.1)***	0.9 (0.5–1.8)	4.3 (0.7–26.8)

Each line corresponds to a different multivariate model. The dependent variable is the perceived usefulness of the corresponding treatment. Independent variables are, for each model, gender, age, training in CM, and personal use of CM. For example: Personal use of CM was associated with higher perceived usefulness of osteopathic treatments. Missing values were handled by multiple imputation. Treatments are classified from most often considered useful to least often considered useful.

CM = complementary medicine; NSAIDs = non-steroidal anti-inflammatory drugs

* P<0.05

** P<0.01

*** P<0.001

† Reference group = male

‡ Reference group = no CM training

§ Reference group = <56 years

I Reference group = no personal use of CM

¶ For conventional treatments, personal use of any CM; For CM treatments, personal use of that specific CM

### Reported prescribing behavior for conventional treatments

Treatments most prescribed by PCPs were physiotherapy (99.2% [95% CI, 98.0–99.7%]), NSAIDs (97.4% [95% CI, 95.6–98.4%]), and acetaminophen (94.4% [95% CI, 92.1–96.1%]). Treatments that were less prescribed were opioids (89.1% [95% CI, 86.1–91.5%]), manual therapy (80.3% [95% CI, 76.7–83.5%]), and chiropractic (71.8% [95% CI, 67.8–75.5%]). However, the number of patients to whom these conventional treatments were prescribed varied greatly. Each of the top 3 most prescribed treatments was prescribed by approximatively one third to a half of the PCPs to more than 75% of their patients, whereas more than two thirds of PCPs prescribed treatments such as opioids or spinal/nerve blocks to less than 26% of their patients (Table 3).

**Table 3:** PCPs’ prescription frequency of treatments for chronic or recurrent LBP.

	For each treatment, distribution of PCPs % (95% CI) according to the proportion of patients to whom they prescribe or recommend that treatment (upper quartile, inter quartile, lower quartile, none)			
Proportion of patients receiving each treatment	76–100%	26–75%	1–25%	0%
Treatment prescribed or recommended				
Acetaminophen	50.5 (46.2–54.7)	33.2 (29.4–37.4)	10.7 (8.4–13.7)	5.6 (3.9–7.9)
Physiotherapy	45.1 (40.0–49.3)	45.1 (40.0–49.3)	9.1 (6.9–11.8)	0.8 (0.3–2.0)
NSAIDs	31.7 (27.9–35.8)	51.0 (46.7–55.2)	14.7 (11.9–17.9)	2.6 (1.6–4.4)
Muscle relaxants	12.2 (9.7–15.3)	41.9 (37.8–46.2)	36.4 (32.4–40.6)	9.5 (7.3–12.3)
Manual therapy	11.1 (8.7–14.1)	28.8 (25.1–32.8)	40.4 (36.3–44.7)	19.7 (16.5–23.2)
Osteopathic treatment	8.8 (6.6–11.6)	33.9 (30.0–38.1)	44.5 (40.3–48.8)	12.7 (10.1–15.9)
Acupuncture	3.4 (2.1–5.4)	13.5 (10.8–16.7)	52.4 (48.1–56.6)	30.7 (26.9–34.8)
Chiropractic	2.8 (1.7–4.6)	17.5 (14.5–20.9)	51.6 (47.3–55.8)	28.2 (24.5–32.2)
Opioids	1.6 (0.8–3.1)	16.4 (13.4–19.8)	71.1 (67.1–74.8)	10.9 (8.5–13.9)
Spinal/nerve blocks	1.5 (0.7–3.0)	17.8 (14.7–21.3)	71.6 (67.6–75.2)	9.1 (6.9–11.9)

Missing values were handled by multiple imputation.

chronic or recurrent LBP = chronic or recurrent low back pain; PCPs = primary care physicians; NSAIDs = nonsteroidal anti-inflammatory drugs.

In a multivariate analysis, we did not observe a clear influence of gender, age or personal use of CM on reported prescribing behavior of conventional treatments. However, there was a tendency for PCPs with CM training to recommend conventional treatments less often than PCPs without such training.

### Perceived usefulness of CM

For CM in general, 71.7% [95% CI, 67.7–75.3%] of the respondents considered it to be useful: in a multivariate analysis women were more convinced than men (OR = 1.8 [95% CI, 1.1–3.0], P = 0.03) and older physicians (≥56 years) perceived it as being less useful than younger physicians did (OR = 0.3 [95% CI, 0.2–0.5], P<0.001). Furthermore, training in CM or the personal use of CM were both associated with higher perceived usefulness of CM in general (OR = 1.9 [95% CI, 1.0–3.4], P = 0.03; and OR = 4.8 [95% CI, 3.0–7.6], P<0.001, respectively).

The top 3 CMs perceived as useful for chronic or recurrent LBP were osteopathic treatment (78.4% [95% CI, 74.7–81.7%]), yoga (69.3% [95% CI, 65.2–73.1%]), and therapeutic massage (63.9% [95% CI, 59.7–67.9%]). Multivariate analysis showed that there was a tendency among female PCPs, and PCPs trained in CM or using CM to consider more often CM treatments useful. We did not observe such a tendency for PCPs’ age (Table 2).

### Recommendation of CM

Overall, 2.1% ([95% CI, 1.2–3.7%]) of PCPs never recommended any CM for the treatment chronic or recurrent LBP. In a multivariate analysis, women were more likely than men to recommend CM in general (OR = 2.1 [95% CI, 1.1–4.0], P = 0.03). Older PCPs (≥56 years) were less likely to recommend CM in general than were younger PCPs (OR = 0.3 [95% CI, 0.2–0.5], P<0.001). Furthermore, personal use of CM was significantly associated with recommendation of CM in general (OR = 4.8 [95% CI, 2.7–8.7], P<0.001).

Osteopathic treatment (87.3% [95% CI, 84.1–89.9%]), acupuncture (69.3% [95% CI, 65.2–73.1%]), and therapeutic massage (58.7% [95% CI, 54.4–62.9%]) were the 3 most recommended CMs. In a multivariate analysis, there was a tendency among female PCPs, younger PCPs and PCPs trained in CM to recommend CM treatments more than other PCPs. In addition, self-use of a given CM was associated with more recommendation for that particular CM (Table 4).

**Table 4:** Multivariate regression models of prescription of conventional treatments and recommendation for complementary medicine treatments.

Treatments	Gender	CM training	Age	Personal use of CM ^¶^
	Female^†^	Yes^‡^	≥56 years^§^	Yes^I^
	OR (95% CI)	OR (95% CI)	OR (95% CI)	OR (95% CI)
**Conventional treatments**				
Physiotherapy	1.7 (0.2–18.3)	0.1 (0.0–1.2)	0.4 (0.0–4.6)	1.4 (0.1–17.1)
NSAIDs	0.6 (0.2–2.0)	0.2 (0.1–0.7)*	0.9 (0.3–2.9)	0.7 (0.2–3.0)
Acetaminophen	0.5 (0.2–1.2)	0.3 (0.1–0.7)**	0.4 (0.2–1.0)	0.7 (0.3–1.8)
Spinal/nerve blocks	0.7 (0.4–1.3)	0.5 (0.3–1.0)	1.0 (0.5–2.0)	0.6 (0.3–1.3)
Muscle relaxants	1.3 (0.7–2.5)	0.6 (0.3–1.1)	0.7 (0.4–1.4)	0.8 (0.4–1.6)
Opioids	0.8 (0.4–1.4)	0.2 (0.1–0.4)***	0.5 (0.3–0.9)*	1.1 (0.6–2.0)
Manual therapy	1.5 (0.9–2.5)	0.8 (0.4–1.3)	0.9 (0.6–1.5)	1.7 (1.1–2.7)*
Chiropractic	0.7 (0.4–1.0)	0.9 (0.6–1.5)	1.4 (0.9–2.2)	1.1 (0.7–1.7)
**Complementary medicine**				
Osteopathic treatment	1.6 (0.8–3.2)	1.4 (0.7–3.0)	0.4 (0.2–0.8)**	8.1 (2.8–23.0)***
Acupuncture	1.9 (1.2–3.0)**	2.4 (1.4–4.1)**	0.8 (0.5–1.2)	2.3 (1.3–4.2)**
Therapeutic massage	0.8 (0.6–1.3)	1.2 (0.7–1.8)	1.3 (0.9–1.9)	7.9 (3.7–17.1) ***
Yoga	1.6 (1.1–2.3)*	1.1 (0.7–1.7)	1.2 (0.8–1.7)	13.5 (4.1–44.5)***
Sophrology	1.3 (0.9–2.0)	0.9 (0.6–1.4)	1.5 (1.0–2.1)*	6.4 (2.9–14.3)***
Hypnosis	1.6 (1.1–2.4)*	2.1 (1.4–3.3)***	0.7 (0.5–1.0)	4.1 (2.0–8.1)***
Meditation	1.7 (1.1–2.5)*	1.7 (1.1–2.7)*	0.7 (0.5–1.1)	5.9 (3.1–11.3)***
Herbal medicine	1.2 (0.8–1.8)	1.9 (1.2–3.0)**	1.1 (0.7–1.7)	3.5 (2.1–5.9)***
Tai chi	1.5 (1.0–2.4)	1.4 (0.9–2.3)	1.0 (0.7–1.6)	18.8 (6.4–55.8)***
Shiatsu	1.7 (1.1–2.7)*	1.7 (1.1–2.7)*	1.4 (0.9–2.2)	8.0 (2.9–21.6)***
Traditional healing	0.7 (0.4–1.1)	1.3 (0.8–2.1)	1.7 (1.1–2.7)*	6.5 (2.6–15.9)***
Kinesiology	1.0 (0.6–1.6)	1.2 (0.7–2.0)	1.3 (0.8–2.1)	6.4 (2.5–16.4)***
Homeopathy	1.6 (1.0–2.7)	2.7 (1.5–4.6)***	2.2 (1.3–3.9)**	6.6 (3.7–11.9)***
Reflexology	1.2 (0.7–2.0)	1.5 (0.9–2.6)	1.5 (0.9–2.5)	6.7 (3.1–14.4)***
Reiki	1.4 (0.7–2.8)	1.1 (0.5–2.3)	1.2 (0.6–2.4)	7.2 (2.8–18.6)***
Magnetism	1.6 (0.8–3.4)	1.2 (0.5–2.5)	2.3 (1.1–5.0)*	27.2 (10.1–73.5)***
Aromatherapy	1.4 (0.7–3.0)	2.8 (1.3–5.9)**	1.2 (0.6–2.6)	10.1 (4.3–23.7)***
Art-therapy	1.7 (0.9–3.3)	1.3 (0.6–2.8)	1.8 (0.9–3.5)	9.0 (1.6–51.3)*
Ayurvedic medicine	2.9 (1.3–6.4)**	0.8 (0.3–1.9)	2.1 (0.9–4.6)	28.1 (9.5–82.9)***
Anthroposophic medicine	2.8 (1.1–7.1)*	1.9 (0.8–4.6)	1.9 (0.8–4.7)	16.0 (2.9–87.5)**
Chinese herbs	1.5 (0.5–3.9)	0.8 (0.3–2.2)	3.0 (1.1–8.1)*	23.0 (6.4–83.1)***

Each line corresponds to a different multivariate model. The dependent variable is the prescription or recommendation of the corresponding treatment. Independent variables are, for each model, gender, age, training in CM, and personal use of CM. For example: Personal use of CM was associated with higher perceived usefulness of osteopathic treatments. Missing values were handled by multiple imputation. Treatments are classified from most often prescribed/recommended to least often prescribed/recommended.

CM = complementary medicine; NSAIDs = nonsteroidal anti-inflammatory drugs

* P <0.05

** P <0.01

*** P <0.001

† Reference group = male

‡ Reference group = no CM training

§ Reference group = <56 years

I Reference group = no personal use of CM

¶ For conventional treatments, personal use of any CM; For CM treatments, personal use of that specific CM

Finally, 34.6% ([95% CI, 30.7–38.8%]) of the respondents stated that they did not usually recommend CM to patients because they did not know any reliable therapists and 31.0% ([95% CI, 27.2–35.1%]) because they did not have enough knowledge about CM.

### Comparison between conventional medicine and CM

Most conventional treatments showed higher rates of prescription than of perceived usefulness, whereas the opposite scenario was observed for most CMs, with the exception of osteopathic treatment and acupuncture. Overall, with the exception of osteopathic treatment, all conventional treatments were more often considered useful than CM treatments (Fig 2). This tendency was confirmed for individual reported prescription behavior. Results showed a significant association between PCPs’ perceived usefulness and their reported prescribing behavior (*P* values of chi-squared tests all <0.001). However, the strength of the correlation (phi coefficient) was weak, which can be explained by the fact that some PCPs prescribed treatments that they did not consider useful and did not prescribe treatment they found useful. The strength of the correlation for conventional and complementary treatments is shown in S1 Table.

## Discussion

This study showed that the top three conventional treatments most often considered useful in the management of chronic or recurrent LBP were physiotherapy, NSAIDs, and manual therapy, whereas the most prescribed conventional treatments were physiotherapy, NSAIDs, and acetaminophen. Osteopathic treatment, yoga, and therapeutic massage were the most often considered useful CMs in the management of chronic or recurrent LBP. The most recommended CM treatments were osteopathic treatment, acupuncture, and therapeutic massage.

Overall, our study showed an association between the perceived usefulness of various treatment options for chronic or recurrent LBP and PCPs’ reported prescription or recommendation behaviors. However, the correlation between individual perceived usefulness and reported prescriptions/recommendations was not strong. Results indicated that PCPs tended to prescribe or recommend treatments that they did not perceive as being very useful (mostly conventional medications) and not to prescribe or recommend treatments they found useful (mostly CM). Although such discrepancies between perceived usefulness and prescription behavior have also been identified in the management of acute low back pain, no further investigations of the reasons have so far been conducted [45]. However, numerous studies have investigated factors influencing management of low back pain by PCPs. Factors commonly mentioned that could explain the discrepancies found in our study were patient preferences [31, 46], patient pressure or perceived pressure to obtain medication [33, 35], preservation of the patient-physician relationship [28, 35, 46, 47], PCPs’ own professional and personal experiences [28, 31, 32, 48], and physicians’ beliefs regarding low back pain [25, 37, 49, 50]. More specifically, our results showed that a significant number of PCPs prescribed some conventional treatments (opioids, spinal/nerve blocks, and muscle relaxants) although they did not consider them to be as useful as other less prescribed treatments (such as manual therapy and osteopathic treatment). The reasons for this may be found in the factors outlined above. Another factor associated with prescribing treatments that were not perceived as being very useful might be the ease with which PCPs could prescribe drugs rather than other treatments. Drugs are immediately available, whereas referral to specialists for other treatments may imply a delay in patient care [32, 34, 51], even more so when treatments are not readily available or cannot be reimbursed by the conventional medical system [36]. However, these observations must be balanced by the fact that some treatments (such as opioids or spinal/nerve blocks) seem to have been prescribed by many PCPs, but to a small number of patients. These results highlight the importance of better understanding the prescribing patterns of PCPs in the context of chronic or recurrent LBP and unveiling reasons associated with low recommendation of some treatments despite scientific data.

Physiotherapy, NSAIDs, and acetaminophen were among the 3 most recommended treatments, which is consistent with what most guidelines used to recommend for first-line treatment in chronic low back pain [3, 17], but inconsistent with the most recent guideline released by the American College of Physicians, which recommends non-pharmacologic treatments as first-line treatment [19]. However, as these guidelines were published after data collection for this study, we cannot make a comment on the potential endorsement of these guidelines by PCPs or not. One study suggested that this high use of drugs might be related to physicians treating chronic pain as if it were acute pain [26].

The top 4 CMs most often perceived as being useful were also the most recommended (Osteopathic treatment, acupuncture, yoga, and therapeutic massage). Interestingly, all four CM treatments have shown some effect in the treatment of chronic or recurrent LBP [9, 14, 16]. These treatments, among others, were already recommended in the ACP’s 2007 guidelines as second-line treatments [18] and appear in the 2017 guidelines as first-line options [19].

A majority of PCPs seemed to be familiar with the few CM treatments that can be recommended for chronic or recurrent LBP. However, our results showed that a significant number of PCPs did not usually recommend CM because they did not know any reliable CM provider and hence did not know where to direct their patients. This problem was also highlighted by PCPs in a recent study about management of LBP in primary care in England [32]. Overall, few studies investigated PCPs’ recommendation for CM in the context of LBP. However, some hypotheses can be formulated regarding the relatively low frequency of CM recommendations by PCPs. First, the fact that in Switzerland, most CM treatments are not covered by basic health insurance might discourage PCPs from recommending them to their patients [36]. Second, as most CM practitioners in Switzerland are legally not considered part of the medical profession, PCPs might fear malpractice litigations and thus choose not to recommend such treatments to their patients with LBP [48].

Women were more likely than men to consider CM useful and to recommend CM treatments. Other studies have shown that female physicians were more likely to recommend CM treatments than were male physicians [40, 52]. Overall, physicians’ age was not strongly associated with perceived usefulness or recommendation of CM. However, PCPs younger than 56 years considered CM in general to be more useful than did older PCPs. Being younger was also associated with more CM recommendations in general. This finding may indicate better acceptance and better knowledge of CM among younger generations of PCPs whose professional activity evolved alongside the increase in CM use in the general population. In our study, physicians trained in at least one CM were more likely to consider CM as being useful and to recommend CM than were physicians who were not trained in CM. Similarly, Corbin and Shapiro [52] found that physicians’ belief in specific CM efficacy was related to education in that CM modality and that physicians’ education about specific modalities were clearly associated with a high frequency of physician recommendation of that modality to the patient. Moreover, they found that physicians who used CM were almost 7 times more likely to recommend CM to their patients than were physicians who did not use CM [52]. Our results also showed a strong correlation between self-use of CM and perceived usefulness or recommendation of CM in general.

To our knowledge, this study is the first in years to investigate PCPs perceived usefulness of chronic or recurrent LBP treatments and their reported prescription/recommendation behaviors. It is also the first to investigate about many different therapies recommended by PCPs for their chronic or recurrent LBP patients. Moreover, our results are important as our study happened in a period of shift about recommendations for LBP treatments. This study has several limitations. First, there may have been a selection bias in the sense that PCPs interested in pain management and CM may have participated more than others. Second, our study did not investigate the frequency of prescription of one particular treatment but rather the number of PCPs having already prescribed this treatment at least once. Third, our study did not investigate the number of patients to whom CM treatments were recommended. Fourth, the population studied might not be representative of PCPs from other countries. However, our PCPs practiced medicine within a population base of about 2 million residents, living in urban and countryside settings. Finally, as it was an exploratory study, we made many statistical comparisons and some differences or associations may have occurred by chance.

## Conclusion

Non-pharmacologic treatments for the management of chronic or recurrent LBP, including CM, seem to be underprescribed in comparison with pharmacological treatments. Considering the frequency and burden of chronic or recurrent LBP, the most (cost-) effective treatments should be proposed to patients. Implementation programs targeting PCPs should be used, as well as better access to and reimbursement for effective non-pharmacologic treatments. Repeating the study in a near future would permit to see if the recommendation of the recent guidelines will be followed by PCPs.

## Supporting information

S1 Table Strength of correlation between PCPs’ perceived usefulness of conventional treatments and reported prescribing behavior. Results of columns 2 to 5 are expressed as a number of participants, omitting missing values. The phi coefficient (column 6) indicates for each treatment the strength of the correlation between the perceived usefulness and the reported prescription behavior. The computation of the phi coefficient is based on the multiple imputations.
(PDF)

S1 File Study questionnaire (English version). (PDF)

S2 File Study questionnaire (French version). (PDF)
