# Supplementary material for: Primary care physicians’ attitude and reported prescribing behavior for chronic low back pain: An exploratory cross-sectional study
Source: PLoS One. 2018 Sep 27;13(9):e0204613. doi: 10.1371/journal.pone.0204613 (PMC6160127; doi:10.1371/journal.pone.0204613)
Supplement: S1 Table — Results of columns 2 to 5 are expressed as a number of participants, omitting missing values. The phi coefficient (column 6) indicates for each treatment the strength of the correlation between the perceived usefulness and the reported prescription behavior. The computation of the phi coefficient is based on the multiple imputations. (PDF) [file pone.0204613.s001.pdf]

| Treatment | Not useful / not recommended | Useful / not recommended | Not useful / recommended | Useful / recommended | phi (95%CI) |
|---|---|---|---|---|---|
| Opioids | 45 | 12 | 107 | 362 | 0.39 (0.31-0.46) |
| Spinal/nerve blocks | 33 | 14 | 83 | 394 | 0.35 (0.27-0.43) |
| Acetaminophen | 28 | 1 | 65 | 437 | 0.50 (0.43-0.56) |
| NSAIDs | 11 | 2 | 50 | 466 | 0.37 (0.29-0.45) |
| Muscle relaxants | 46 | 4 | 86 | 390 | 0.49 (0.42-0.55) |
| Manual therapy | 52 | 51 | 37 | 385 | 0.43 (0.36-0.50) |
| Physiotherapy | 4 | 0 | 21 | 502 | 0.38 (0.30-0.46) |
| Chiropractic | 107 | 41 | 60 | 315 | 0.54 (0.47-0.60) |
| **Complementary medicine (in general)** | 76 | 12 | 66 | 367 | 0.59 (0.53-0.65) |
| Osteopathic treatment | 52 | 14 | 56 | 401 | 0.53 (0.46-0.59) |
| Acupuncture | 129 | 31 | 73 | 287 | 0.56 (0.50-0.62) |
| Aromatherapy | 470 | 17 | 9 | 22 | 0.60 (0.52-0.66) |
| Art-therapy | 418 | 67 | 7 | 27 | 0.38 (0.30-0.46) |
| Hypnosis | 221 | 122 | 16 | 167 | 0.53 (0.47-0.59) |
| Homeopathy | 412 | 25 | 20 | 69 | 0.71 (0.65-0.75) |
| Therapeutic massage | 148 | 67 | 38 | 271 | 0.58 (0.52-0.63) |
| Anthroposophic medicine | 473 | 28 | 2 | 15 | 0.43 (0.34-0.52) |
| Ayurvedic medicine | 459 | 37 | 3 | 23 | 0.49 (0.41-0.57) |
| Chinese herbs | 468 | 39 | 3 | 12 | 0.41 (0.31-0.50) |
| Meditation | 224 | 128 | 12 | 163 | 0.53 (0.47-0.59) |
| Magnetism | 461 | 26 | 10 | 25 | 0.56 (0.49-0.62) |
| Traditional healing | 351 | 65 | 29 | 79 | 0.52 (0.45-0.58) |
| Shiatsu | 311 | 93 | 11 | 108 | 0.56 (0.50-0.62) |
| Reflexology | 380 | 62 | 12 | 71 | 0.58 (0.52-0.64) |
| Sophrology | 220 | 90 | 17 | 200 | 0.62 (0.56-0.67) |
| Tai-chi | 280 | 109 | 9 | 125 | 0.55 (0.49-0.61) |
| Herbal medicine | 344 | 34 | 24 | 124 | 0.73 (0.68-0.77) |
| Yoga | 134 | 100 | 22 | 266 | 0.53 (0.47-0.59) |
| Kinesiology | 376 | 50 | 16 | 72 | 0.57 (0.50-0.63) |
| Reiki | 430 | 49 | 5 | 32 | 0.50 (0.43-0.57) |
