# Supplementary material for: Primary care physicians’ attitude and reported prescribing behavior for chronic low back pain: An exploratory cross-sectional study
Source: PLoS One. 2018 Sep 27;13(9):e0204613. doi: 10.1371/journal.pone.0204613 (PMC6160127; doi:10.1371/journal.pone.0204613)
Supplement: S1 File — (PDF) [file pone.0204613.s002.pdf]

Institut universitaire de médecine sociale et préventive
Biopôle 2 - Route de la Corniche 10
CH-1010 Lausanne

Institut universitaire de médecine de famille iumf
Faculté de biologie et de médecine
Université de Lausanne

Service d'anesthésiologie
Centre d'antalgie
Bâtiment hospitalier - 07.700
Bugnon 46
CH-1011 Lausanne

Questionnaire for primary care physicians

Treatment of chronic low back pain in primary care

Many patients with chronic or recurrent low back pain use complementary therapies. We wish to know your opinion about complementary medicines in the treatment of such conditions.

DEFINITIONS:

Definitions used in this study :

**Chronic**	Low back pain (LBP) lasting for three months or more
and / or	
**recurrent low back pain**	At least two episodes or more of LBP during the past 12 month, with a significant impact on the patient's daily life (for example: frequent need to move, difficulty walking up or down stairs, sleep disorder due to low back pain, inability to get to work, etc.)

Chronic and /or recurrent low back pain can be specific (eg, infection, tumour, fracture, fracture, inflammatory disorder, radicular syndrome) or non specific (low back pain not attributable to a recognisable, known specific pathology).

**Complementary medicine (WHO)**	**CM** refers to a broad set of health care practices that are not part of that country's own tradition or conventional medicine and are not fully integrated into the dominant health-care system<br><br>*In this questionnaire, some therapies such as hypnosis or music therapy are also considered complementary medicines because they are not systematically integrated into the Swiss health system*

We thank you for answering the following questions:

A. SOCIODEMOGRAPHIC CHARACTERISTICS

1. Gender : ☐ Female ☐ Male

2. Age :
☐ ≤ 35 years old
☐ 36-45 years old
☐ 46-55 years old
☐ ≥ 56 years old

3. Country of birth : ____

4. Nationality(-ies) :____

5. For how long have you been working in a medical practice? ________ year(s)

6. Are you trained in one or several complementary therapy (-ies) : ☐ Yes ☐ No

a. If yes :

Which training in complementary medicine have you attended to? *:*	Do you have an official diploma in this method?
☐ Homeopathy	☐ Yes ☐ No
☐ Anthroposophic medicine	☐ Yes ☐ No
☐ Herbal medicine	☐ Yes ☐ No
☐ Traditional Chinese Medicine and/or acupuncture	☐ Yes ☐ No
☐ Neural therapy	☐ Yes ☐ No
☐ Hypnosis	☐ Yes ☐ No
☐ Manual medicine	☐ Yes ☐ No
☐ Ayurvedic medicine	☐ Yes ☐ No
☐ Other (s): __________________________ ___________________________________	☐ Yes ☐ No

7. Which title-s of medicine do you hold (several possible answers*):*
☐ Practicing physician
☐ General internal medicine or internal medicine or general medicine
☐ Other federal title (s) (FMH) :________________________________

8. How many half days do you work in your medical practice?
0- 1 – 2 – 3 – 4 – 5 – 6 – 7 – 8 – 9 – 10

9. In which canton (s) do you practice? ____________________________________

B. PHARMACOLOGICAL TREATMENT IN THE MANAGEMENT OF CHRONIC OR RECURRENT LOW BACK PAIN

1. Do you think opioids, regardless to the galenic form, may be useful for the treatment of patients with chronic or recurrent low back pain?
☐ Strongly agree
☐ Agree
☐ Neither agree nor disagree
☐ Disagree
☐ Strongly disagree

2. To which percentage of your patients do you prescribe **opioids**, regardless of the dosage form, for the treatment of chronic or recurrent low back pain?

☐ 0%
☐ 1-25%
☐ 26-50%
☐ 51-75%
☐ 76-99%
☐ 100%

3. Do you think that **interventional blocks** may be useful for the treatment of patients with chronic or recurrent low back pain?

☐ Strongly agree
☐ Agree
☐ Neither agree nor disagree
☐ Disagree
☐ Strongly disagree

4. To which percentage of your patients do you prescribe **interventional blocks** for the treatment of patients with chronic or recurrent low back pain?

☐ 0%
☐ 1-25%
☐ 26-50%
☐ 51-75%
☐ 76-99%
☐ 100%

5. Do you think that **acetaminophen** may prove useful for the treatment of patients with chronic or recurrent low back pain?

☐ Strongly agree
☐ Agree
☐ Neither agree nor disagree
☐ Disagree
☐ Strongly disagree

6. To which percentage of your patients do you prescribe **acetaminophen** for the treatment of patients with chronic or recurrent low back pain?

☐ 0%
☐ 1-25%
☐ 26-50%
☐ 51-75%
☐ 76-99%
☐ 100%

7. Do you think that **NSAIDS** may prove useful for the treatment of patients with chronic or recurrent low back pain?

☐ Strongly agree
☐ Agree
☐ Neither agree nor disagree
☐ Disagree
☐ Pas du tout d'accord

8. To which percentage of your patients do you prescribe **NSAIDS** for the treatment of patients with chronic or recurrent low back pain?

☐ 0%
☐ 1-25%
☐ 26-50%
☐ 51-75%
☐ 76-99%
☐ 100%

9. Do you think that **muscle relaxants** may prove useful for the treatment of patients with chronic or recurrent low back pain?

☐ Strongly agree
☐ Agree
☐ Neither agree nor disagree
☐ Disagree
☐ Strongly disagree

10. To which percentage of your patients do you prescribe **muscle relaxants** for the treatment of patients with chronic or recurrent low back pain?

☐ 0%
☐ 1-25%
☐ 26-50%
☐ 51-75%
☐ 76-99%
☐ 100%

11. Do you think that la **manual medicine** may prove useful for the treatment of patients with chronic or recurrent low back pain?

☐ Strongly agree
☐ Agree
☐ Neither agree nor disagree
☐ Disagree
☐ Strongly disagree

12. To which percentage of your patients do you prescribe **manual medicine** for the treatment of patients with chronic or recurrent low back pain?

☐ 0%
☐ 1-25%
☐ 26-50%
☐ 51-75%
☐ 76-99%
☐ 100%

13. Do you think that **physiotherapy** may prove useful for the treatment of patients with chronic or recurrent low back pain?

☐ Strongly agree
☐ Agree
☐ Neither agree nor disagree
☐ Disagree
☐ Strongly disagree

14. To which percentage of your patients do you prescribe **physiotherapy** for the treatment of patients with chronic or recurrent low back pain?

☐ 0%
☐ 1-25%
☐ 26-50%
☐ 51-75%
☐ 76-99%
☐ 100%

15. Do you think that **chiropractic** (manipulative therapy exercised by a chiropractor) may prove useful for the treatment of patients with chronic or recurrent low back pain?

☐ Strongly agree
☐ Agree
☐ Neither agree nor disagree
☐ Disagree
☐ Strongly disagree

16. To which percentage of your patients do you prescribe **chiropractic** for the treatment of patients with chronic or recurrent low back pain?
- ☐ 0%
- ☐ 1-25%
- ☐ 26-50%
- ☐ 51-75%
- ☐ 76-99%
- ☐ 100%

C. COMPLEMENTARY MEDICINE TREATMENTS IN THE MANAGEMENT OF CHRONIC OR RECURRENT LOW BACK PAIN

1. Do you think that some **complementary medicine** may prove useful for the treatment of patients with chronic or recurrent low back pain?
- ☐ Strongly agree
- ☐ Agree
- ☐ Neither agree nor disagree
- ☐ Disagree
- ☐ Strongly disagree

2. To which percentage of your patients do you recommend **complementary medicine** in general for the treatment of patients with chronic or recurrent low back pain?
- ☐ 0%
- ☐ 1-25%
- ☐ 26-50%
- ☐ 51-75%
- ☐ 76-99%
- ☐ 100%

3. Do you think that **osteopathic treatment** may prove useful for the treatment of patients with chronic or recurrent low back pain?
- ☐ Strongly agree
- ☐ Agree
- ☐ Neither agree nor disagree
- ☐ Disagree
- ☐ Strongly disagree
- ☐ I am unfamiliar with this approach

4. To which percentage of your patients do you recommend **osteopathic treatment** for the treatment of patients with chronic or recurrent low back pain?
- ☐ 0%
- ☐ 1-25%
- ☐ 26-50%
- ☐ 51-75%
- ☐ 76-99%
- ☐ 100%

5. Do you think that **acupuncture** may prove useful for the treatment of patients with chronic or recurrent low back pain?
- ☐ Strongly agree
- ☐ Agree
- ☐ Neither agree nor disagree
- ☐ Disagree
- ☐ Strongly disagree
- ☐ I am unfamiliar with this approach

6. To which percentage of your patients do you recommend **acupuncture** for the treatment of patients with chronic or recurrent low back pain?
- ☐ 0%
- ☐ 1-25%
- ☐ 26-50%
- ☐ 51-75%
- ☐ 76-99%
- ☐ 100%

7. Do you think that **aromatherapy** (essential oils) may prove useful for the treatment of patients with chronic or recurrent low back pain?
- ☐ Strongly agree
- ☐ Agree
- ☐ Neither agree nor disagree
- ☐ Disagree
- ☐ Strongly disagree
- ☐ I am unfamiliar with this approach

8. Have you ever recommended **aromatherapy** for the treatment of patients with chronic or recurrent low back pain? (at least once)
- ☐ Yes
- ☐ No

9. Do you think that **aromatherapy** may prove useful for the treatment of patients with chronic or recurrent low back pain?
- ☐ Strongly agree
- ☐ Agree
- ☐ Neither agree nor disagree
- ☐ Disagree
- ☐ Strongly disagree
- ☐ I am unfamiliar with this approach

10. Have you ever recommended **art-therapy** for the treatment of patients with chronic or recurrent low back pain? (at least once)
- ☐ Yes
- ☐ No

11. Do you think that **hypnosis** may prove useful for the treatment of patients with chronic or recurrent low back pain?
- ☐ Strongly agree
- ☐ Agree
- ☐ Neither agree nor disagree
- ☐ Disagree
- ☐ Strongly disagree
- ☐ I am unfamiliar with this approach

12. Have you ever recommended **hypnosis** for the treatment of patients with chronic or recurrent low back pain? (at least once)
- ☐ Yes
- ☐ No

13. Do you think that **homeopathy** may prove useful for the treatment of patients with chronic or recurrent low back pain?
- ☐ Strongly agree
- ☐ Agree
- ☐ Neither agree nor disagree
- ☐ Disagree
- ☐ Strongly disagree
- ☐ I am unfamiliar with this approach

14. Have you ever recommended de **homeopathy** for the treatment of patients with chronic or recurrent low back pain? (at least once)
- ☐ Yes
- ☐ No

15. Do you think that **therapeutic massage** may prove useful for the treatment of patients with chronic or recurrent low back pain?
- ☐ Strongly agree
- ☐ Agree
- ☐ Neither agree nor disagree
- ☐ Disagree
- ☐ Strongly disagree
- ☐ I am unfamiliar with this approach

16. Have you ever recommended **therapeutic massage** for the treatment of patients with chronic or recurrent low back pain? (at least once)
- ☐ Yes
- ☐ No

17. Do you think that la **anthroposophic medicine** may prove useful for the treatment of patients with chronic or recurrent low back pain?
- ☐ Strongly agree
- ☐ Agree
- ☐ Neither agree nor disagree
- ☐ Disagree
- ☐ Strongly disagree
- ☐ I am unfamiliar with this approach

18. Have you ever recommended **anthroposophic medicine** for the treatment of patients with chronic or recurrent low back pain? (at least once)
- ☐ Yes
- ☐ No

19. Do you think that **ayurvedic medicine** may prove useful for the treatment of patients with chronic or recurrent low back pain?
- ☐ Strongly agree
- ☐ Agree
- ☐ Neither agree nor disagree
- ☐ Disagree
- ☐ Strongly disagree
- ☐ I am unfamiliar with this approach

20. Have you ever recommended **ayurvedic medicine** for the treatment of patients with chronic or recurrent low back pain? (at least once)
- ☐ Yes
- ☐ No

21. Do you think that **Chinese herbs** (as a part of Traditional Chinese Medicine) may prove useful for the treatment of patients with chronic or recurrent low back pain?
- ☐ Strongly agree
- ☐ Agree
- ☐ Neither agree nor disagree
- ☐ Disagree
- ☐ Strongly disagree
- ☐ I am unfamiliar with this approach

22. Have you ever recommended des **Chinese herbs** (as a part of Traditional Chinese Medicine) for the treatment of patients with chronic or recurrent low back pain? (at least once)
- ☐ Yes
- ☐ No

23. Do you think that **meditation** may prove useful for the treatment of patients with chronic or recurrent low back pain?
- ☐ Strongly agree
- ☐ Agree
- ☐ Neither agree nor disagree
- ☐ Disagree
- ☐ Strongly disagree
- ☐ I am unfamiliar with this approach

24. Have you ever recommended **meditation** for the treatment of patients with chronic or recurrent low back pain? (at least once)
- ☐ Yes
- ☐ No

25. Do you think that **magnetism** may prove useful for the treatment of patients with chronic or recurrent low back pain?
- ☐ Strongly agree
- ☐ Agree
- ☐ Neither agree nor disagree
- ☐ Disagree
- ☐ Strongly disagree
- ☐ I am unfamiliar with this approach

26. Have you ever recommended **magnetism** for the treatment of patients with chronic or recurrent low back pain? (at least once)
- ☐ Yes
- ☐ No

27. Do you think that traditional healers may prove useful for the treatment of patients with chronic or recurrent low back pain?
- ☐ Strongly agree
- ☐ Agree
- ☐ Neither agree nor disagree
- ☐ Disagree
- ☐ Strongly disagree
- ☐ I am unfamiliar with this approach

28. Have you ever recommended medicine healers for the treatment of patients with chronic or recurrent low back pain? (at least once)
- ☐ Yes
- ☐ No

29. Do you think that **shiatsu** may prove useful for the treatment of patients with chronic or recurrent low back pain?
- ☐ Strongly agree
- ☐ Agree
- ☐ Neither agree nor disagree
- ☐ Disagree
- ☐ Strongly disagree
- ☐ I am unfamiliar with this approach

30. Have you ever recommended **shiatsu** for the treatment of patients with chronic or recurrent low back pain? (at least once)
- ☐ Yes
- ☐ No

31. Do you think that **reflexology** (reflexotherapy) may prove useful for the treatment of patients with chronic or recurrent low back pain?
- ☐ Strongly agree
- ☐ Agree
- ☐ Neither agree nor disagree
- ☐ Disagree
- ☐ Strongly disagree
- ☐ I am unfamiliar with this approach

32. Have you ever recommended **reflexology** for the treatment of patients with chronic or recurrent low back pain? (at least once)
- ☐ Yes
- ☐ No

33. Do you think that **sophrology** may prove useful for the treatment of patients with chronic or recurrent low back pain?
- ☐ Strongly agree
- ☐ Agree
- ☐ Neither agree nor disagree
- ☐ Disagree
- ☐ Strongly disagree
- ☐ I am unfamiliar with this approach

34. Have you ever recommended **sophrology** for the treatment of patients with chronic or recurrent low back pain? (at least once)
- ☐ Yes
- ☐ No

35. Do you think that **tai chi/chi gong** may prove useful for the treatment of patients with chronic or recurrent low back pain?
- ☐ Strongly agree
- ☐ Agree
- ☐ Neither agree nor disagree
- ☐ Disagree
- ☐ Strongly disagree
- ☐ I am unfamiliar with this approach

36. Have you ever recommended **tai chi/chi gong** for the treatment of patients with chronic or recurrent low back pain? (at least once)
- ☐ Yes
- ☐ No

37. Do you think that **herbal medicine** may prove useful for the treatment of patients with chronic or recurrent low back pain?
- ☐ Strongly agree
- ☐ Agree
- ☐ Neither agree nor disagree
- ☐ Disagree
- ☐ Strongly disagree
- ☐ I am unfamiliar with this approach

38. Have you ever recommended **herbal medicine** for the treatment of patients with chronic or recurrent low back pain? (at least once)
- ☐ Yes
- ☐ No

39. Do you think that **yoga** may prove useful for the treatment of patients with chronic or recurrent low back pain?
- ☐ Strongly agree
- ☐ Agree
- ☐ Neither agree nor disagree
- ☐ Disagree
- ☐ Strongly disagree
- ☐ I am unfamiliar with this approach

40. Have you ever recommended **yoga** for the treatment of patients with chronic or recurrent low back pain? (at least once)
- ☐ Yes
- ☐ No

41. Do you think that **kinesiology** may prove useful for the treatment of patients with chronic or recurrent low back pain?
- ☐ Strongly agree
- ☐ Agree
- ☐ Neither agree nor disagree
- ☐ Disagree
- ☐ Strongly disagree
- ☐ I am unfamiliar with this approach

42. Have you ever recommended **kinesiology** for the treatment of patients with chronic or recurrent low back pain? (at least once)
- ☐ Yes
- ☐ No

43. Do you think that **reiki** may prove useful for the treatment of patients with chronic or recurrent low back pain?
- ☐ Strongly agree
- ☐ Agree
- ☐ Neither agree nor disagree
- ☐ Disagree
- ☐ Strongly disagree
- ☐ I am unfamiliar with this approach

44. Have you ever recommended **reiki** for the treatment of patients with chronic or recurrent low back pain? (at least once)
- ☐ Yes
- ☐ No

45. Do you know any <u>other</u> complementary medicine, which may prove useful for the treatment of patients with chronic or recurrent low back pain?
- ☐ Yes → if yes, which one-s: ____
- ☐ No

46. Have you ever recommended **another complementary medicine** for the treatment of patients with chronic or recurrent low back pain? (at least once)
- ☐ Yes → if yes, which one-s: ____
- ☐ No

47. Among <u>your patients</u> with chronic or recurrent low back pain, which percentage do you think **uses** complementary medicine for the management of their low back pain (of their own choice or recommended by a doctor)?
- ☐ 0%
- ☐ 1-25%
- ☐ 26-50%
- ☐ 51-75%
- ☐ 76-99%
- ☐ 100%

48. Among <u>your patients</u> with chronic or recurrent low back pain, which percentage do you think **informs you** by their use of complementary medicine for the management of their low back pain (spontaneously or in response to your question)?

- ☐ 0%
- ☐ 1-25%
- ☐ 26-50%
- ☐ 51-75%
- ☐ 76-99%
- ☐ 100%

D. ATTITUDE TOWARDS COMPLEMENTARY MEDICINE IN GENERAL

NOT ONLY FOR CHRONIC OR RECURRENT LOW BACK PAIN

1. During a conversation with a patient about complementary medicine, who initiates the conversation usually?

- ☐ You
- ☐ The patient
- ☐ About 50/50 between you and the patient
- ☐ I never / hardly ever talk about complementary medicine with my patients

2. To which percentage of your patients do you discuss about the **benefits** of complementary medicine?

- ☐ 0%
- ☐ 1-25%
- ☐ 26-50%
- ☐ 51-75%
- ☐ 76-99%
- ☐ 100%

3. To which percentage of your patients do you discuss about the **harms** of complementary medicine?

- ☐ 0%
- ☐ 1-25%
- ☐ 26-50%
- ☐ 51-75%
- ☐ 76-99%
- ☐ 100%

4. Do you think that your knowledge is sufficient to inform your patients about complementary medicine?

- ☐ Strongly agree
- ☐ Agree
- ☐ Neither agree nor disagree
- ☐ Disagree
- ☐ Strongly disagree

5. What do you think of the following statements?

a) Physicians should have **basic knowledge** of the most common complementary medicines.

- ☐ Strongly agree
- ☐ Agree
- ☐ Neither agree nor disagree
- ☐ Disagree
- ☐ Strongly disagree

b) Complementary medicine offer a fair cost / effectiveness ratio.

- ☐ Strongly agree
- ☐ Agree
- ☐ Neither agree nor disagree
- ☐ Disagree
- ☐ Strongly disagree

c) Health professionals should be able to **inform patients** about complementary medicine.

- ☐ Strongly agree

☐ Agree
☐ Neither agree nor disagree
☐ Disagree
☐ Strongly disagree

d) There is a need for further **scientific research** on complementary medicine*.*
☐ Strongly agree
☐ Agree
☐ Neither agree nor disagree
☐ Disagree
☐ Strongly disagree

e) I lack information about complementary medicine
☐ Strongly agree
☐ Agree
☐ Neither agree nor disagree
☐ Disagree
☐ Strongly disagree

6. I <u>do not recommend</u> complementary medicine, because I do not know any reliable therapists and therefore do not know where to direct my patients for these therapies.
☐ Strongly agree
☐ Agree
☐ Neither agree nor disagree
☐ Disagree
☐ Strongly disagree

7. <u>During your life</u>, have you ever used, for yourself, complementary medicine therapy-ies for own health problems in general?
☐ No
☐ Do not wish to answer this question
☐ Yes

If yes, specify which one-s :

☐ Acupuncture
☐ Aromatherapy / essential oils
☐ Art-therapy
☐ Hypnosis
☐ Homeopathy
☐ Therapeutic massage
☐ Anthroposophic
☐ Ayurvedic medicine
☐ Chinese herbs (part of Traditional Chinese Medicine)
☐ Meditation
☐ Osteopathic treatment
☐ Magnetism
☐ Traditional healing
☐ Shiatsu
☐ Reflexotherapy (Reflexology)
☐ Sophrology
☐ Tai chi and/or qi gong
☐ Herbal medicine
☐ Yoga
☐ Kinesioloy
☐ Reiki
☐ Other *(specify)* : ______________________

8. Do you take into consideration health insurance coverage plan (having a supplementary insurance covering complementary medicines) before referring your patients to a complementary medicine therapist?
☐ Never
☐ Rarely
☐ Sometimes
☐ Often
☐ Always

Do you have any comments on complementary medicine?

Do you have any comments on the questionnaire?

Please slide the completed questionnaire into the stamped envelope and mail it.

***** Thank you for answering this questionnaire *****
