# Supplementary material for: Primary care physicians’ attitude and reported prescribing behavior for chronic low back pain: An exploratory cross-sectional study
Source: PLoS One. 2018 Sep 27;13(9):e0204613. doi: 10.1371/journal.pone.0204613 (PMC6160127; doi:10.1371/journal.pone.0204613)
Supplement: S2 File — (PDF) [file pone.0204613.s003.pdf]

Institut universitaire de médecine sociale et préventive
Biopôle 2 - Route de la Corniche 10
CH-1010 Lausanne

Institut universitaire de médecine de famille
Faculté de biologie et de médecine
Université de Lausanne

Service d'anesthésiologie
Centre d'antalgie
Bâtiment hospitalier - 07.700
Bugnon 46
CH-1011 Lausanne

QUESTIONNAIRE À L'INTENTION DES MÉDECINS DE FAMILLE

Etude au cabinet du médecin de famille sur l'utilisation des médecines complémentaires par les patients présentant des lombalgies chroniques ou récurrentes

De nombreux patients souffrant de lombalgies chroniques ou récurrentes ont recours à des thérapies complémentaires. Nous souhaitons connaître votre opinion sur les médecines complémentaires dans le traitement des lombalgies chroniques ou récurrentes.

Il faut compter environ 10 minutes pour remplir le questionnaire.

DÉFINITIONS:

Définitions utilisées dans le cadre de cette étude :

**Lombalgies chroniques**	3 mois ou plus
**et/ ou**	
**Lombalgies récurrentes**	Minimum 2 épisodes durant les 12 derniers mois<br>Dont la douleur a eu un impact sur la vie quotidienne (comme par exemple : besoin fréquent de bouger, difficulté à monter ou descendre les escaliers, trouble du sommeil dû à la lombalgie, incapacité à se rendre au travail,…)

Les lombalgies chroniques ou récurrentes peuvent être d'étiologie **spécifique** (hernie discale, fracture, spondylolisthésis, ostéoporose,…) ou **non spécifique** (d'étiologie inconnue : musculaire, posturale, ergonomique,…).

**Médecines complémentaires (OMS)**	Large ensemble de pratiques de soins qui ne font pas partie de la tradition (académique) du pays ou qui ne sont pas intégrées dans le système de santé dominant (par exemple en Suisse : l'acupuncture, l'ostéopathie, l'homéopathie…).<br>*Dans ce questionnaire, certaines thérapies comme l'hypnose ou la musicothérapie sont aussi considérées comme des médecines complémentaires car elles ne sont pas intégrées de manière systématique dans le système de santé suisse.*

CONFIDENTIALITÉ :

Toutes les réponses sont anonymes.

CONTACT:

Pour toute question ou commentaire, vous pouvez appeler ou écrire à Mme Anne-Sylvie Bill:

✉ **anne-sylvie.bill@chuv.ch** ou ✆ **079 556 44 38** (les mardis, jeudis, vendredis : 9h -17h)

Nous vous remercions de répondre aux questions suivantes :

A. DONNÉES SOCIODÉMOGRAPHIQUES VOUS CONCERNANT

1. Genre : ☐ Femme ☐ Homme

2. Tranche d'âge :
☐ ≤ 35 ans
☐ 36-45 ans
☐ 46-55 ans
☐ ≥ 56 ans

3. Pays de naissance : ____

4. Nationalité(s) :____

5. Depuis combien de temps êtes-vous installé(e) en cabinet ? ________ année(s)

Avez-vous suivi une formation dans la pratique d'une (ou plusieurs) médecine(s) complémentaire(s) : ☐ Oui ☐ Non

a. Si oui :

Quelle(s) formation(s) en médecine complémentaire avez-vous suivie(s) :	Êtes-vous au bénéfice d'une AFC pour la pratique de cette méthode ?
☐ Homéopathie	☐ Oui ☐ Non
☐ Médecine anthroposophique	☐ Oui ☐ Non
☐ Phytothérapie	☐ Oui ☐ Non
☐ Médecine traditionnelle chinoise et/ou acupuncture	☐ Oui ☐ Non
☐ Thérapie neurale	☐ Oui ☐ Non
☐ Hypnose	☐ Oui ☐ Non
☐ Médecine manuelle	☐ Oui ☐ Non
☐ Médecine ayurvédique	☐ Oui ☐ Non
☐ Autres: ___________________________ ___________________________________	☐ Oui ☐ Non

6. Quel(s) titre(s) de médecin possédez-vous (plusieurs réponses possibles):
☐ Médecin praticien-ne
☐ Médecine interne générale ou médecin interne ou médecine générale
☐ Autre titre FMH :________________________________

7. Quel est le nombre de demi-journées où vous consultez au cabinet ?
Entourer le chiffre qui convient
0- 1 – 2 – 3 – 4 – 5 – 6 – 7 – 8 – 9 – 10

8. Dans quel(s) canton(s) exercez vous ? _____________________________________

B. LES TRAITEMENTS MÉDICAMENTEUX DANS LA PRISE EN CHARGE DES LOMBALGIES CHRONIQUES OU RÉCURRENTES

1. Pensez-vous que les **opiacés,** quelle que soit la forme galénique, puissent se révéler utiles dans le traitement des patient-e-s présentant des lombalgies chroniques ou récurrentes ?
☐ Tout à fait d'accord
☐ D'accord
☐ Ni d'accord, ni pas d'accord
☐ Pas d'accord
☐ Pas du tout d'accord

2. A quel pourcentage de vos patient-e-s prescrivez-vous des **opiacés**, quelle que soit la forme galénique, pour le traitement des lombalgies chroniques ou récurrentes ?
   - ☐ 0%
   - ☐ 1-25%
   - ☐ 26-50%
   - ☐ 51-75%
   - ☐ 76-99%
   - ☐ 100%

3. Pensez-vous que **l'antalgie interventionnelle** puisse se révéler utile dans le traitement des patient-e-s présentant des lombalgies chroniques ou récurrentes ?
   - ☐ Tout à fait d'accord
   - ☐ D'accord
   - ☐ Ni d'accord, ni pas d'accord
   - ☐ Pas d'accord
   - ☐ Pas du tout d'accord

4. A quel pourcentage de vos patient-e-s prescrivez-vous de **l'antalgie interventionnelle** pour le traitement des lombalgies chroniques ou récurrentes ?
   - ☐ 0%
   - ☐ 1-25%
   - ☐ 26-50%
   - ☐ 51-75%
   - ☐ 76-99%
   - ☐ 100%

5. Pensez-vous que le **paracétamol** puisse se révéler utile dans le traitement des patient-e-s présentant des lombalgies chroniques ou récurrentes ?
   - ☐ Tout à fait d'accord
   - ☐ D'accord
   - ☐ Ni d'accord, ni pas d'accord
   - ☐ Pas d'accord
   - ☐ Pas du tout d'accord

6. A quel pourcentage de vos patient-e-s prescrivez-vous du **paracétamol** pour le traitement des lombalgies chroniques ou récurrentes ?
   - ☐ 0%
   - ☐ 1-25%
   - ☐ 26-50%
   - ☐ 51-75%
   - ☐ 76-99%
   - ☐ 100%

7. Pensez-vous que les **AINS** puissent se révéler utiles dans le traitement des patient-e-s présentant des lombalgies chroniques ou récurrentes ?
   - ☐ Tout à fait d'accord
   - ☐ D'accord
   - ☐ Ni d'accord, ni pas d'accord
   - ☐ Pas d'accord
   - ☐ Pas du tout d'accord

8. A quel pourcentage de vos patient-e-s prescrivez-vous des **AINS** pour le traitement des lombalgies chroniques ou récurrentes ?
   - ☐ 0%
   - ☐ 1-25%
   - ☐ 26-50%
   - ☐ 51-75%
   - ☐ 76-99%

☐ 100%

9. Pensez-vous que les **myorelaxants** puissent se révéler utiles dans le traitement des patient-e-s présentant des lombalgies chroniques ou récurrentes ?
   ☐ Tout à fait d'accord
   ☐ D'accord
   ☐ Ni d'accord, ni pas d'accord
   ☐ Pas d'accord
   ☐ Pas du tout d'accord

10. A quel pourcentage de vos patient-e-s <u>prescrivez-vous</u> **des myorelaxants** pour le traitement des lombalgies chroniques ou récurrentes ?
   ☐ 0%
   ☐ 1-25%
   ☐ 26-50%
   ☐ 51-75%
   ☐ 76-99%
   ☐ 100%

11. Pensez-vous que la **médecine manuelle** puisse se révéler utile dans le traitement des patient-e-s présentant des lombalgies chroniques ou récurrentes ?
   ☐ Tout à fait d'accord
   ☐ D'accord
   ☐ Ni d'accord, ni pas d'accord
   ☐ Pas d'accord
   ☐ Pas du tout d'accord

12. A quel pourcentage de vos patient-e-s <u>prescrivez-vous</u> de la **médecine manuelle** pour le traitement des lombalgies chroniques ou récurrentes ?
   ☐ 0%
   ☐ 1-25%
   ☐ 26-50%
   ☐ 51-75%
   ☐ 76-99%
   ☐ 100%

13. Pensez-vous que la **physiothérapie** puisse se révéler utile dans le traitement des patient-e-s présentant des lombalgies chroniques ou récurrentes ?
   ☐ Tout à fait d'accord
   ☐ D'accord
   ☐ Ni d'accord, ni pas d'accord
   ☐ Pas d'accord
   ☐ Pas du tout d'accord

14. A quel pourcentage de vos patient-e-s <u>prescrivez-vous</u> de **la physiothérapie** pour le traitement des lombalgies chroniques ou récurrentes ?
   ☐ 0%
   ☐ 1-25%
   ☐ 26-50%
   ☐ 51-75%
   ☐ 76-99%
   ☐ 100%

15. Pensez-vous que la **chiropraxie** (thérapie manuelle exercée par un chiropracticien) puisse se révéler utile dans le traitement des patient-e-s présentant des lombalgies chroniques ou récurrentes ?
   ☐ Tout à fait d'accord
   ☐ D'accord
   ☐ Ni d'accord, ni pas d'accord
   ☐ Pas d'accord
   ☐ Pas du tout d'accord

16. A quel pourcentage de vos patient-e-s <u>prescrivez-vous</u> de la **chiropraxie** pour le traitement des lombalgies chroniques ou récurrentes ?

- ☐ 0%
- ☐ 1-25%
- ☐ 26-50%
- ☐ 51-75%
- ☐ 76-99%
- ☐ 100%

C. LES MÉDECINES COMPLÉMENTAIRES DANS LA PRISE EN CHARGE DES LOMBALGIES CHRONIQUES OU RÉCURRENTES

1. Pensez-vous que certaines médecines complémentaires puissent se révéler utiles dans le traitement des patient-e-s présentant des lombalgies chroniques ou récurrentes ?

- ☐ Tout à fait d'accord
- ☐ D'accord
- ☐ Ni d'accord, ni pas d'accord
- ☐ Pas d'accord
- ☐ Pas du tout d'accord

2. A quel pourcentage de vos patient-e-s <u>recommandez-vous</u> des médecines complémentaires en général pour le traitement des lombalgies chroniques ou récurrentes ?

- ☐ 0%
- ☐ 1-25%
- ☐ 26-50%
- ☐ 51-75%
- ☐ 76-99%
- ☐ 100%

3. Pensez-vous que l'**ostéopathie** puisse être utile dans le traitement des patient-e-s présentant des lombalgies chroniques ou récurrentes ?

- ☐ Tout à fait d'accord
- ☐ D'accord
- ☐ Ni d'accord, ni pas d'accord
- ☐ Pas d'accord
- ☐ Pas du tout d'accord
- ☐ Je ne connais pas cette approche

4. A quel pourcentage de vos patient-e-s <u>recommandez-vous</u> de l'**ostéopathie** pour le traitement des lombalgies chroniques ou récurrentes ?

- ☐ 0%
- ☐ 1-25%
- ☐ 26-50%
- ☐ 51-75%
- ☐ 76-99%
- ☐ 100%

5. Pensez-vous que l'**acupuncture** puisse être utile dans le traitement des patient-e-s présentant des lombalgies chroniques ou récurrentes ?

- ☐ Tout à fait d'accord
- ☐ D'accord
- ☐ Ni d'accord, ni pas d'accord
- ☐ Pas d'accord
- ☐ Pas du tout d'accord
- ☐ Je ne connais pas cette approche

6. A quel pourcentage de vos patient-e-s recommandez-vous de l'**acupuncture** pour le traitement des lombalgies chroniques ou récurrentes ?
   - ☐ 0%
   - ☐ 1-25%
   - ☐ 26-50%
   - ☐ 51-75%
   - ☐ 76-99%
   - ☐ 100%

7. Pensez-vous que l'**aromathérapie** (huiles essentielles) puisse être utile dans le traitement des patient-e-s présentant des lombalgies chroniques ou récurrentes ?
   - ☐ Tout à fait d'accord
   - ☐ D'accord
   - ☐ Ni d'accord, ni pas d'accord
   - ☐ Pas d'accord
   - ☐ Pas du tout d'accord
   - ☐ Je ne connais pas cette approche

8. Avez-vous déjà recommandé de l'**aromathérapie** pour le traitement des patient-e-s présentant des lombalgies chroniques ou récurrentes ? (au moins une fois)
   - ☐ Oui
   - ☐ Non

9. Pensez-vous que l'**art-thérapie** puisse être utile dans le traitement des patient-e-s présentant des lombalgies chroniques ou récurrentes ?
   - ☐ Tout à fait d'accord
   - ☐ D'accord
   - ☐ Ni d'accord, ni pas d'accord
   - ☐ Pas d'accord
   - ☐ Pas du tout d'accord
   - ☐ Je ne connais pas cette approche

10. Avez-vous déjà recommandé de l'**art-thérapie** pour le traitement des patient-e-s présentant des lombalgies chroniques ou récurrentes ? (au moins une fois)
   - ☐ Oui
   - ☐ Non

11. Pensez-vous que l'**hypnose** puisse être utile dans le traitement des patient-e-s présentant des lombalgies chroniques ou récurrentes ?
   - ☐ Tout à fait d'accord
   - ☐ D'accord
   - ☐ Ni d'accord, ni pas d'accord
   - ☐ Pas d'accord
   - ☐ Pas du tout d'accord
   - ☐ Je ne connais pas cette approche

12. Avez-vous déjà recommandé de l'**hypnose** pour le traitement des patient-e-s présentant des lombalgies chroniques ou récurrentes ? (au moins une fois)
   - ☐ Oui
   - ☐ Non

13. Pensez-vous que l'**homéopathie** puisse être utile dans le traitement des patient-e-s présentant des lombalgies chroniques ou récurrentes ?
   - ☐ Tout à fait d'accord
   - ☐ D'accord
   - ☐ Ni d'accord, ni pas d'accord
   - ☐ Pas d'accord
   - ☐ Pas du tout d'accord
   - ☐ Je ne connais pas cette approche

14. Avez-vous déjà recommandé de l'**homéopathie** pour le traitement des patient-e-s présentant des lombalgies chroniques ou récurrentes ? (au moins une fois)
- ☐ Oui
- ☐ Non

15. Pensez-vous que le **massage thérapeutique** puisse être utile dans le traitement des patient-e-s présentant des lombalgies chroniques ou récurrentes ?
- ☐ Tout à fait d'accord
- ☐ D'accord
- ☐ Ni d'accord, ni pas d'accord
- ☐ Pas d'accord
- ☐ Pas du tout d'accord
- ☐ Je ne connais pas cette approche

16. Avez-vous déjà recommandé le **massage thérapeutique** pour le traitement des patient-e-s présentant des lombalgies chroniques ou récurrentes ? (au moins une fois)
- ☐ Oui
- ☐ Non

17. Pensez-vous que la **médecine anthroposophique** puisse être utile dans le traitement des patient-e-s présentant des lombalgies chroniques ou récurrentes ?
- ☐ Tout à fait d'accord
- ☐ D'accord
- ☐ Ni d'accord, ni pas d'accord
- ☐ Pas d'accord
- ☐ Pas du tout d'accord
- ☐ Je ne connais pas cette approche

18. Avez-vous déjà recommandé de la **médecine anthroposophique** pour le traitement des patient-e-s présentant des lombalgies chroniques ou récurrentes ? (au moins une fois)
- ☐ Oui
- ☐ Non

19. Pensez-vous que la **médecine ayurvédique** puisse être utile dans le traitement des patient-e-s présentant des lombalgies chroniques ou récurrentes ?
- ☐ Tout à fait d'accord
- ☐ D'accord
- ☐ Ni d'accord, ni pas d'accord
- ☐ Pas d'accord
- ☐ Pas du tout d'accord
- ☐ Je ne connais pas cette approche

20. Avez-vous déjà recommandé de la **médecine ayurvédique** pour le traitement des patient-e-s présentant des lombalgies chroniques ou récurrentes ? (au moins une fois)
- ☐ Oui
- ☐ Non

21. Pensez-vous que les **herbes chinoises** (issues de la médecine chinoise) puissent être utiles dans le traitement des patient-e-s présentant des lombalgies chroniques ou récurrentes ?
- ☐ Tout à fait d'accord
- ☐ D'accord
- ☐ Ni d'accord, ni pas d'accord
- ☐ Pas d'accord
- ☐ Pas du tout d'accord
- ☐ Je ne connais pas cette approche

22. Avez-vous déjà recommandé des **herbes chinoises** (issues de la médecine chinoise) pour le traitement des patient-e-s présentant des lombalgies chroniques ou récurrentes ? (au moins une fois)
- ☐ Oui
- ☐ Non

23. Pensez-vous que la **méditation** puisse être utile dans le traitement des patient-e-s présentant des lombalgies chroniques ou récurrentes ?

☐ Tout à fait d'accord
☐ D'accord
☐ Ni d'accord, ni pas d'accord
☐ Pas d'accord
☐ Pas du tout d'accord
☐ Je ne connais pas cette approche

24. Avez-vous déjà recommandé de la **méditation** pour le traitement des patient-e-s présentant des lombalgies chroniques ou récurrentes ? (au moins une fois)

☐ Oui
☐ Non

25. Pensez-vous que le **magnétisme** puisse être utile dans le traitement des patient-e-s présentant des lombalgies chroniques ou récurrentes ?

☐ Tout à fait d'accord
☐ D'accord
☐ Ni d'accord, ni pas d'accord
☐ Pas d'accord
☐ Pas du tout d'accord
☐ Je ne connais pas cette approche

26. Avez-vous déjà recommandé du **magnétisme** pour le traitement des patient-e-s présentant des lombalgies chroniques ou récurrentes ? (au moins une fois)

☐ Oui
☐ Non

27. Pensez-vous que la **reboutologie** (rebouteux) puisse être utile dans le traitement des patient-e-s présentant des lombalgies chroniques ou récurrentes ?

☐ Tout à fait d'accord
☐ D'accord
☐ Ni d'accord, ni pas d'accord
☐ Pas d'accord
☐ Pas du tout d'accord
☐ Je ne connais pas cette approche

28. Avez-vous déjà recommandé de la **reboutologie** pour le traitement des patient-e-s présentant des lombalgies chroniques ou récurrentes ? (au moins une fois)

☐ Oui
☐ Non

29. Pensez-vous que le **shiatsu** puisse être utile dans le traitement des patient-e-s présentant des lombalgies chroniques ou récurrentes ?

☐ Tout à fait d'accord
☐ D'accord
☐ Ni d'accord, ni pas d'accord
☐ Pas d'accord
☐ Pas du tout d'accord
☐ Je ne connais pas cette approche

30. Avez-vous déjà recommandé du **shiatsu** pour le traitement des patient-e-s présentant des lombalgies chroniques ou récurrentes ? (au moins une fois)

☐ Oui
☐ Non

31. Pensez-vous que la **réflexologie** (réflexothérapie) puisse être utile dans le traitement des patient-e-s présentant des lombalgies chroniques ou récurrentes ?

☐ Tout à fait d'accord
☐ D'accord
☐ Ni d'accord, ni pas d'accord
☐ Pas d'accord
☐ Pas du tout d'accord
☐ Je ne connais pas cette approche

32. Avez-vous déjà recommandé de la **réflexologie** pour le traitement des patient-e-s présentant des lombalgies chroniques ou récurrentes ? (au moins une fois)

☐ Oui
☐ Non

33. Pensez-vous que la **sophrologie** puisse être utile dans le traitement des patient-e-s présentant des lombalgies chroniques ou récurrentes ?

☐ Tout à fait d'accord
☐ D'accord
☐ Ni d'accord, ni pas d'accord
☐ Pas d'accord
☐ Pas du tout d'accord
☐ Je ne connais pas cette approche

34. Avez-vous déjà recommandé de la **sophrologie** pour le traitement des patient-e-s présentant des lombalgies chroniques ou récurrentes ? (au moins une fois)

☐ Oui
☐ Non

35. Pensez-vous que le **tai chi/chi gong** puisse être utile dans le traitement des patient-e-s présentant des lombalgies chroniques ou récurrentes ?

☐ Tout à fait d'accord
☐ D'accord
☐ Ni d'accord, ni pas d'accord
☐ Pas d'accord
☐ Pas du tout d'accord
☐ Je ne connais pas cette approche

36. Avez-vous déjà recommandé du **tai chi/chi gong** pour le traitement des patient-e-s présentant des lombalgies chroniques ou récurrentes ? (au moins une fois)

☐ Oui
☐ Non

37. Pensez-vous que la **phytothérapie** puisse être utile dans le traitement des patient-e-s présentant des lombalgies chroniques ou récurrentes ?

☐ Tout à fait d'accord
☐ D'accord
☐ Ni d'accord, ni pas d'accord
☐ Pas d'accord
☐ Pas du tout d'accord
☐ Je ne connais pas cette approche

38. Avez-vous déjà recommandé de la **phytothérapie** pour le traitement des patient-e-s présentant des lombalgies chroniques ou récurrentes ? (au moins une fois)

☐ Oui
☐ Non

39. Pensez-vous que le **yoga** puisse être utile dans le traitement des patient-e-s présentant des lombalgies chroniques ou récurrentes ?
- ☐ Tout à fait d'accord
- ☐ D'accord
- ☐ Ni d'accord, ni pas d'accord
- ☐ Pas d'accord
- ☐ Pas du tout d'accord
- ☐ Je ne connais pas cette approche

40. Avez-vous déjà recommandé du **yoga** pour le traitement des patient-e-s présentant des lombalgies chroniques ou récurrentes ? (au moins une fois)
- ☐ Oui
- ☐ Non

41. Pensez-vous que la **kinésiologie** puisse être utile dans le traitement des patient-e-s présentant des lombalgies chroniques ou récurrentes ?
- ☐ Tout à fait d'accord
- ☐ D'accord
- ☐ Ni d'accord, ni pas d'accord
- ☐ Pas d'accord
- ☐ Pas du tout d'accord
- ☐ Je ne connais pas cette approche

42. Avez-vous déjà recommandé de la **kinésiologie** pour le traitement des patient-e-s présentant des lombalgies chroniques ou récurrentes ? (au moins une fois)
- ☐ Oui
- ☐ Non

43. Pensez-vous que le **reiki** puisse être utile dans le traitement des patient-e-s présentant des lombalgies chroniques ou récurrentes ?
- ☐ Tout à fait d'accord
- ☐ D'accord
- ☐ Ni d'accord, ni pas d'accord
- ☐ Pas d'accord
- ☐ Pas du tout d'accord
- ☐ Je ne connais pas cette approche

44. Avez-vous déjà recommandé du **reiki** pour le traitement des patient-e-s présentant des lombalgies chroniques ou récurrentes ? (au moins une fois)
- ☐ Oui
- ☐ Non

45. Connaissez-vous une **autre méthode** de médecine complémentaire qui puisse être utile dans le traitement des patient-e-s présentant des lombalgies chroniques ou récurrentes ?
- ☐ Oui → Si oui, laquelle/lesquelles : ____
- ☐ Non

46. Avez-vous déjà recommandé une **autre méthode** de médecine complémentaire pour le traitement des patient-e-s présentant des lombalgies chroniques ou récurrentes ? (au moins une fois)
- ☐ Oui → Si oui, laquelle/lesquelles : ____
- ☐ Non

47. Parmi vos patient-e-s qui présentent des lombalgies chroniques ou récurrentes, selon vous quel pourcentage utilise des médecines complémentaires pour le traitement de leurs lombalgies (de leur propre choix ou conseillé par un(e) médecin) ?
- ☐ 0%
- ☐ 1-25%
- ☐ 26-50%
- ☐ 51-75%
- ☐ 76-99%
- ☐ 100%

48. Parmi vos patient-e-s qui présentent des lombalgies chroniques ou récurrentes et qui ont recours aux médecines complémentaires, selon vous quel pourcentage **vous informe** (spontanément ou en réponse à vos questions) de leur utilisation des médecines complémentaires ?

☐ 0%
☐ 1-25%
☐ 26-50%
☐ 51-75%
☐ 76-99%
☐ 100%

D. ATTITUDE FACE AUX MÉDECINES COMPLÉMENTAIRES EN GÉNÉRAL

PAS UNIQUEMENT DANS LE CAS DES LOMBALGIES CHRONIQUES OU RÉCURRENTES

1. Lors d'une conversation avec un-e patient-e à propos des médecines complémentaires, qui initie la conversation en général ?

☐ Vous
☐ Le/la patient-e
☐ Environ 50/50 entre vous et le/la patient-e
☐ Je ne parle jamais/très rarement de médecines complémentaires avec mes patient-e-s

2. A quel pourcentage de vos patient-e-s parlez-vous des **bénéfices** des médecines complémentaires ?

☐ 0%
☐ 1-25%
☐ 26-50%
☐ 51-75%
☐ 76-99%
☐ 100%

3. A quel pourcentage de vos patient-e-s parlez-vous des **risques** des médecines complémentaires ?

☐ 0%
☐ 1-25%
☐ 26-50%
☐ 51-75%
☐ 76-99%
☐ 100%

4. Pensez-vous que **vos connaissances** sont suffisantes pour **renseigner** vos patient-e-s au sujet des médecines complémentaires ?

☐ Tout à fait d'accord
☐ D'accord
☐ Ni d'accord, ni pas d'accord
☐ Pas d'accord
☐ Pas du tout d'accord

5. Que pensez-vous des affirmations suivantes ?

a) Les médecins devraient avoir des **connaissances de base** sur les médecines complémentaires les plus connues.

☐ Tout à fait d'accord
☐ D'accord
☐ Ni d'accord, ni pas d'accord
☐ Pas d'accord
☐ Pas du tout d'accord

b) Les médecines complémentaires offrent un bon **rapport efficacité/coût**.

☐ Tout à fait d'accord
☐ D'accord
☐ Ni d'accord, ni pas d'accord
☐ Pas d'accord
☐ Pas du tout d'accord

c) Les professionnels de santé devraient pouvoir **informer les patient-e-s** au sujet des médecines complémentaires.
- ☐ Tout à fait d'accord
- ☐ D'accord
- ☐ Ni d'accord, ni pas d'accord
- ☐ Pas d'accord
- ☐ Pas du tout d'accord

d) Il est nécessaire de développer davantage de **recherche scientifique** sur les médecines complémentaires.
- ☐ Tout à fait d'accord
- ☐ D'accord
- ☐ Ni d'accord, ni pas d'accord
- ☐ Pas d'accord
- ☐ Pas du tout d'accord

e) Je manque d'informations au sujet des médecines complémentaires.
- ☐ Tout à fait d'accord
- ☐ D'accord
- ☐ Ni d'accord, ni pas d'accord
- ☐ Pas d'accord
- ☐ Pas du tout d'accord

6. Je <u>ne recommande pas</u> de **médecines complémentaires**, car je ne connais pas de thérapeutes fiables et ne sais donc pas où orienter mes patient-e-s pour ces approches.
- ☐ Tout à fait d'accord
- ☐ D'accord
- ☐ Ni d'accord, ni pas d'accord
- ☐ Pas d'accord
- ☐ Pas du tout d'accord

7. <u>Au cours de votre vie</u>, avez-vous déjà eu recours, pour vous-même, à une/des médecine(s) complémentaire(s) pour des problèmes de santé en général ?
- ☐ Non
- ☐ Ne souhaite pas répondre
- ☐ Oui

Si oui, spécifiez laquelle/lesquelles :

- ☐ Acupuncture
- ☐ Aromathérapie / huiles essentielles
- ☐ Art-thérapie (Musicothérapie, etc.)
- ☐ Hypnose
- ☐ Homéopathie
- ☐ Massage thérapeutique
- ☐ Médecine anthroposophique
- ☐ Médecine ayurvédique
- ☐ Herbes chinoises (Issues de la médecine chinoise)
- ☐ Méditation
- ☐ Ostéopathie
- ☐ Magnétisme
- ☐ Reboutologie (Rebouteux)
- ☐ Shiatsu
- ☐ Réflexothérapie (Réflexologie)
- ☐ Sophrologie
- ☐ Tai chi et/ou qi gong
- ☐ Phytothérapie (Thérapie par les plantes)
- ☐ Yoga
- ☐ Kinésiologie
- ☐ Reiki
- ☐ Autre *(spécifiez)* : ______________________

8. Prenez-vous en considération le statut assécurologique (le fait d'avoir une assurance couvrant la médecine complémentaire) avant d'adresser vos patients vers un thérapeute complémentaire?
- ☐ Jamais
- ☐ Rarement
- ☐ Parfois
- ☐ Souvent
- ☐ Toujours

Avez-vous des commentaires sur les médecines complémentaires ?

____
____
____
____
____
____
____

Avez-vous des commentaires sur le questionnaire ?

____
____
____
____
____
____

Seriez vous intéressé à participer à d'autres études conduites par l'IUMF ?
Pouvons-nous vous contacter pour de futures études soutenues par l'IUMF ?

- ☐ Oui
- ☐ Non

Veuillez s'il vous plaît glisser le questionnaire rempli dans l'enveloppe affranchie et le poster.

***** Merci d'avoir répondu à ce questionnaire *****
